# Supplementary material for: Genome-Wide Comparison of Structural Variations and Transposon Alterations in Soybean Cultivars Induced by Spaceflight
Source: Int J Mol Sci. 2022 Nov 8;23(22):13721. doi: 10.3390/ijms232213721 (PMC9696660; doi:10.3390/ijms232213721)
Supplement: Supplementary file 1 [file ijms-23-13721-s001.zip › Tables Caption.pdf]

Table S1: Agronomic traits of Z9 and T75 in 2021 and 2022.

Table S2: Differential structural variations were detected between Z9 and T75.

Table S3: Differential transposable elements were detected between Z9 and T75.

Table S4: Differential genes detected between Z9 and T75.
